# Recombinant α- and β-tubulin from *Echinococcus granulosus*: expression, purification and polymerization

**DOI:** 10.1051/parasite/2018063

**Published:** 2018-12-05

**Authors:** Congshan Liu, Jiaqing Yao, Jianhai Yin, Jian Xue, Haobing Zhang

**Affiliations:** 1 National Institute of Parasitic Diseases, Chinese Center for Disease Control and Prevention, Key Laboratory of Parasite and Vector Biology, MOH, National Center for International Research on Tropical Diseases, WHO Collaborating Centre for Tropical Diseases Shanghai 200025 People’s Republic of China

**Keywords:** Microtubule, α- and β-tubulin, *Echinococcus granulosus*, Gene expression, Polymerization assays

## Abstract

Echinococcosis, which causes a high disease burden and is of great public health significance, is caused by the larval stage of *Echinococcus* species. It has been suggested that tubulin is the target of benzimidazoles, the only drugs for the treatment of echinococcosis. This study evaluated the characteristics of tubulins from *Echinococcus granulosus*. The full-length cDNAs of *E. granulosus* α- and β-tubulin isoforms were cloned by reverse transcription PCR from protoscolex RNA. Then, these two tubulin isoforms (α_9_ and β_4_) were recombinantly expressed as insoluble inclusion bodies in *Escherichia coli*. Nickel affinity chromatography was used to purify and refold the contents of these inclusion bodies as active proteins. The polymerization of tubulins was monitored by UV spectrophotometry (A_350_) and confirmed by confocal microscopy and transmission electron microscopy (TEM). Nucleotide sequence analysis revealed that *E. granulosus* 1356 bp α_9_-tubulin and 1332 bp β_4_-tubulin encode corresponding proteins of 451 and 443 amino acids. The average yields of α_9_- and β_4_-tubulin were 2.0–3.0 mg/L and 3.5–5.0 mg/L of culture, respectively. Moreover, recombinant α_9_- and β_4_-tubulin were capable of polymerizing into microtubule-like structures under appropriate conditions *in vitro*. These recombinant tubulins could be helpful for screening anti-*Echinococcus* compounds targeting the tubulins of *E. granulosus*.

## Abstract (fr)

L’échinococcose, une maladie qui revêt une grande importance pour la santé publique, est provoquée par le stade larvaire des espèces d’*Echinococcus*. Il a été suggéré que la tubuline est la cible des benzimidazoles, les seuls médicaments pour le traitement de l’échinococcose. Cette étude a évalué les caractéristiques des tubulines d’*Echinococcus granulosus*. Les ADNc complets des isoformes de tubuline α et β d’*E. granulosus* ont été clonés par PCR par transcription inverse à partir d’ARN de protoscolex. Ensuite, ces deux isoformes de la tubuline (α9 et β4) ont été exprimées par recombinaison sous forme de corps d’inclusion insolubles dans *Escherichia coli*. La chromatographie d’affinité au nickel a été utilisée pour purifier et replier le contenu de ces corps d’inclusion comme protéines actives. La polymérisation des tubulines a été contrôlée par spectrophotométrie UV (A350) et confirmée par microscopie confocale et microscopie électronique à transmission (TEM). L’analyse de la séquence des nucléotides a révélé que la tubuline α9 de 1356 pb et la tubuline β4 de 1332 pb d’*E. granulosus* codent pour des protéines correspondantes de 451 et 443 acides aminés. Les rendements moyens en tubuline α9 et β4 étaient respectivement de 2.0–3.0 mg/L et de 3.5–5.0 mg/L de culture. De plus, les tubulines α9 et β4 recombinantes étaient capables de se polymériser en structures analogues à des microtubules dans des conditions appropriées *in vitro*. Ces tubulines recombinantes pourraient être utiles pour le criblage de composés anti-*Echinococcus* ciblant les tubulines d’*E. granulosus.*

## Introduction

Cystic echinococcosis, which is a global health issue that affects humans and animals, is caused by the metacestode larval stage of *Echinococcus granulosus* [23]. The definitive hosts, intermediate hosts and aberrant intermediate hosts for this parasite are dogs, livestock, and humans, respectively [3, 4]. In livestock and humans, these parasites are mainly located in the liver and lungs [4]. Mebendazole and albendazole, both benzimidazoles (BZs), are drugs for the therapy of echinococcosis [7, 28]. Circumstantial evidence suggests that BZs suppress the polymerization of parasite microtubules (MTs) by binding to the β-tubulin [1, 17], which has made tubulin an attractive target for drug development [27, 39], but studies related to the MTs of *E. granulosus* have been limited.

Microtubules are highly dynamic structures that perform diverse and critical functions in cell structure, cell division, motility, and signal transduction [5, 8]. MTs are composed of soluble tubulin subunits comprising α- and β-tubulins, which are similar in mass (~55 kDa) and share approximately 40% amino acid identity. The formation of MTs reflects the balance between polymerization and de-polymerization of α/β-tubulin heterodimers. The tubulin polymerization assay has already been a powerful tool in characterizing the interactions between drugs and MTs. To date, most functional analyses of MTs have used native tubulins purified from mammalian brain, eukaryotic organisms, kinetoplastid parasites (*Leishmania, Trypanosoma*) and *Saccharomyces cerevisiae* [30]. Although abundant tubulin can be isolated from these sources, the purified proteins are composed of multiple tubulin isoforms and contain only those tubulin subpopulations with assembly competency [35, 37]. Moreover, these results are affected by the other proteins and cofactors that co-purify with native tubulins [27]. In addition, due to the difficulty in collecting enough *E. granulosus* for tubulin extraction, this simple and rapid purification method is not applicable in *E. granulosus* or *E. multilocularis*, which hinders the study of the MTs of this parasite.

Fortunately, there are reports on recombinant human tubulins [37] and helminth tubulins [20, 26] that could polymerize into MTs, indicating that recombinant MTs could be used for high-throughput screening. Hence, based on the previously reported tubulin genes of the parasite and the methods for expressing tubulin and determining the polymerization of the α/β-tubulin heterodimer, we conducted a study on the characteristics of *E. granulosus* tubulin genes and polymerization.

In this study, α- and β-tubulin of *E. granulosus* were expressed in *Escherichia coli* and purified, and these heterodimers were shown to polymerize into microtubule-like structures.

## Materials and methods

### RNA isolation and cDNA synthesis

Protoscoleces were isolated from cysts in the liver of sheep infected with *E. granulosus* (G1 strain, Qinghai, China). Then, total RNA was extracted with the RNeasy Mini Kit (Qiagen, USA) according to the manufacturer’s instructions, followed by reverse transcription using a first-strand cDNA synthesis kit (Toyobo, Japan).

### Sequence amplification of full length α_9_- and β_4_-tubulins

The genes encoding *E. granulosus* α_9_- and β_4_-tubulin were amplified with Ex*Taq* DNA polymerase (Takara, Japan) using gene-specific primers. For α_9_-tubulin, the forward primer was 5′–CGC**GAGCTC**ATGCGTGAATGTATCAGTAT–3′ with a *Sac* I restriction site (in bold), and the reverse primer was 5′–A**GCGGCCGC**TTAGTACTCCTCGCCCTCTT–3′ with a *Not* I restriction site (in bold). For β_4_-tubulin, 5′–CGC**GGATCC**ATGCGAGAGATAGTACACGTT–3′ and 5′–CCC**AAGCTT**TTATGCTTCTTCCTCT–3′ were used as the forward and reverse primers, containing *Bam*H I and *Hin*d III restriction sites (in bold), respectively. The PCR reaction mixture contained 1 μM each primer, 200 μM dNTP mixture, 1× PCR buffer and 0.5 units Ex*Taq* DNA polymerase. PCR conditions were as follows: 5 min at 95 °C for denaturation; 35 cycles of amplification (40 s at 95 °C, 30 s at 60 °C/57 °C for α_9_-tubulin/β_4_-tubulin, 90 s at 72 °C); 10 min at 72 °C for extension. PCR products were separated on 1.2% agarose gels and purified with the Gel Extraction Kit (Qiagen, USA).

### Expression of recombinant proteins

The purified PCR fragments were directly cloned into the pMD19-T vector (Takara, Japan) for TA cloning using the Mighty TA-Cloning Kit (Takara, Japan) and transformed into competent *Escherichia coli* DH5α cells (Tiangen, China), which were incubated at 37 °C overnight on a Luria-Bertani (LB) plate containing 100 μg/mL ampicillin (Sigma, USA). A single clone from each construct was selected and sequenced to ensure sequence fidelity. The verified α_9_- and β_4_-tubulin sequences were cut from the pMD19-T construct by double enzyme digestion and directionally ligated into the pET30a(+) vector (Novagen, USA), which had previously been digested with the same enzymes. Then, plasmid constructs (pET30a-α_9_ and pET30a-β_4_) were confirmed by double enzyme digestion with corresponding enzymes.

The pET30a-α_9_ and pET30a-β_4_ were finally transformed into competent BL21 (DE3) cells (Tiangen, China) using the heat shock method. The positive clones were selected and cultured in 2 L LB medium containing 50 μg/mL kanamycin until the mid-log phase. Expression was induced with 1 mM isopropyl-1-thio-β-D-galactopyranoside (IPTG) for 6 h at 37 °C/200 rpm. The cells were harvested at 8000 × *g* for 15 min, and the pellet was washed with phosphate buffer saline (PBS). The cells were centrifuged again and resuspended in lysis buffer (50 mM Tris-HCl, 300 mM NaCl, 10 mM imidazole, 0.5 mM PMSF, 0.1% Triton X-100, pH 7.4), disrupted by sonication. The inclusion bodies were collected by centrifugation at 12,000 × *g*, 4 °C for 20 min.

### Purification of recombinant proteins

The inclusion bodies were dissolved in binding buffer (50 mM Tris-HCl, 300 mM NaCl, 10 mM imidazole, 8 M urea, pH 7.4), collected by centrifugation at 12,000 × *g* for 20 min at 4 °C and loaded onto an Ni^2+^ Sepharose column (GE Healthcare, USA) pre-equilibrated with the binding buffer. The column was subsequently washed with five column volumes of binding buffer, followed by washing buffer with a linear gradient of urea ranging from 8 M to 0 M. The refolded fusion protein was eluted with elution buffer (50 mM Tris-HCl, 300 mM NaCl, 500 mM imidazole, pH 7.4) and concentrated in an Amicon Ultra centrifugal filter (Millipore, USA). The concentration of recombinant protein was evaluated using a Bradford Kit (Tiangen, China).

### Western blotting

The purified α_9_- or β_4_-tubulin protein was analysed by sodium dodecyl sulfate-polyacrylamide gel electrophoresis (SDS-PAGE) and Western blotting using anti-His antibody (CST, USA, #2366), anti-α-tubulin antibody (CST, USA, #3873) and anti-β-tubulin antibody (CST, USA, #2128) as primary antibodies; the PVDF membrane was blocked for 1 h and then incubated with a 1/1000 dilution of primary antibody at 4 °C for 4 h. The membranes were washed and then incubated with a 1/2500 dilution of anti-mouse (CST, USA, #7076) or anti-rabbit IgG antibody conjugated with HRP (CST, USA, #7074) as a secondary antibody at 4 °C overnight and then washed again. The ECL Kit (Tanon, China) was used to detect the proteins on the PVDF membrane.

### Tubulin polymerization assay

Known concentrations of α_9_- and β_4_-tubulin were diluted with G-PEM buffer (80 mM PIPES, 2 mM MgCl_2_, 0.5 mM EGTA, 1 mM GTP, pH 6.9) to yield final tubulin concentrations of 0.25, 0.5, 1, 2, 3, and 4 mg/mL in a 40 μL reaction mixture. The reaction was carried out at 37 °C, and the OD value was measured at 350 nm every 30 s in a Synergy2 spectrophotometer (Biotek, USA).

### Immunofluorescence and confocal microscopy

The mixture of 2 mg/mL α_9_- and β_4_-tubulin was allowed to polymerize for 1 h at 37 °C and centrifuged at 12,000 × *g* to collect polymerized tubulins. The samples were washed with PBS and centrifuged again. The pellet was fixed in 4% paraformaldehyde at room temperature for 1 h. After washing with PBS five times, the polymerized tubulins were blocked for 1 h at room temperature and incubated with a 1/150 dilution of mouse anti-α-tubulin/Alexa-Fluor 488 antibody (CST, USA, #8058) or anti-β-tubulin/Alexa-Fluor 647 antibody (CST, USA, #3624) at 4 °C overnight. Then, the samples were washed with TBST (1× TBS, 0.1% Tween 20) five times before observation under an A1R-si confocal microscope (Nikon, Japan).

### Transmission electron microscopy (TEM)

Polymerized tubulins were collected for TEM analyses according to the method reported by Vulevic and Correia [38]. In brief, samples were suspended in 100 μL of PEM buffer (80 mM PIPES, 2 mM MgCl_2_, 0.5 mM EGTA, pH 6.9). A total of 30 μL polymerized samples were diluted with 10 μL of 0.4% glutaraldehyde for 1 min at room temperature, and 10 μL of tubulin solution was applied to a 200-mesh, copper/formvar coated grid for 1 min, washed using dH_2_O and stained for 10 min using 1% uranyl acetate. Finally, samples were air dried and viewed with a Tecnai G2 Spirit transmission electron microscope (FEI, USA).

### Sequence analyses and homology modelling

Sequence analyses and alignments were performed using MEGA 6.0 (www.megasoftware.net), BLAST/N (https://blast.ncbi.nlm.nih.gov/Blast.cgi) and Clustal Omega (http://www.ebi.ac.uk/Tools/msa/clustalo/). Then, the homology model of the hetero α_9_- and β_4_-tubulin dimer was generated with HOMCOS (http://homcos.pdbj.org/).

## Results

### Amplification of the α_9_- and β_4_-tubulin genes and plasmid construction

**Figure 1.**
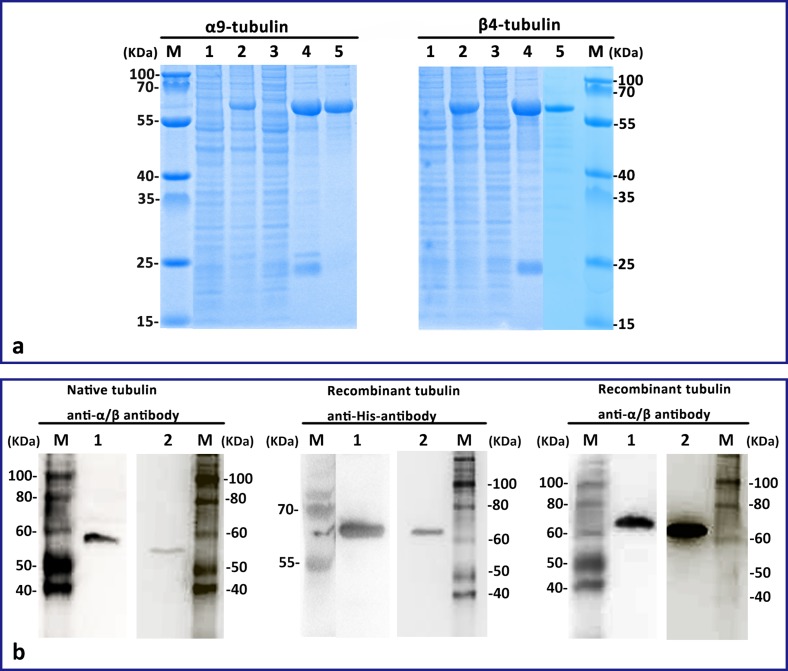
The cloning and expression of α_9_- and β_4_-tubulin. (a) The expression of α_9_- and β_4_-tubulin in *E. coli* BL21 (DE3). M: standard protein molecular weight marker, lane 1: negative control without induction, lane 2: induced control, lane 3: the supernatant after sonication, lane 4: the pellet after sonication, and lane 5: purified recombinant tubulin. (b) Western blot analysis. M: standard protein molecular weight marker, lane 1: α-tubulin, lane 2: β-tubulin.

**Figure 2.**
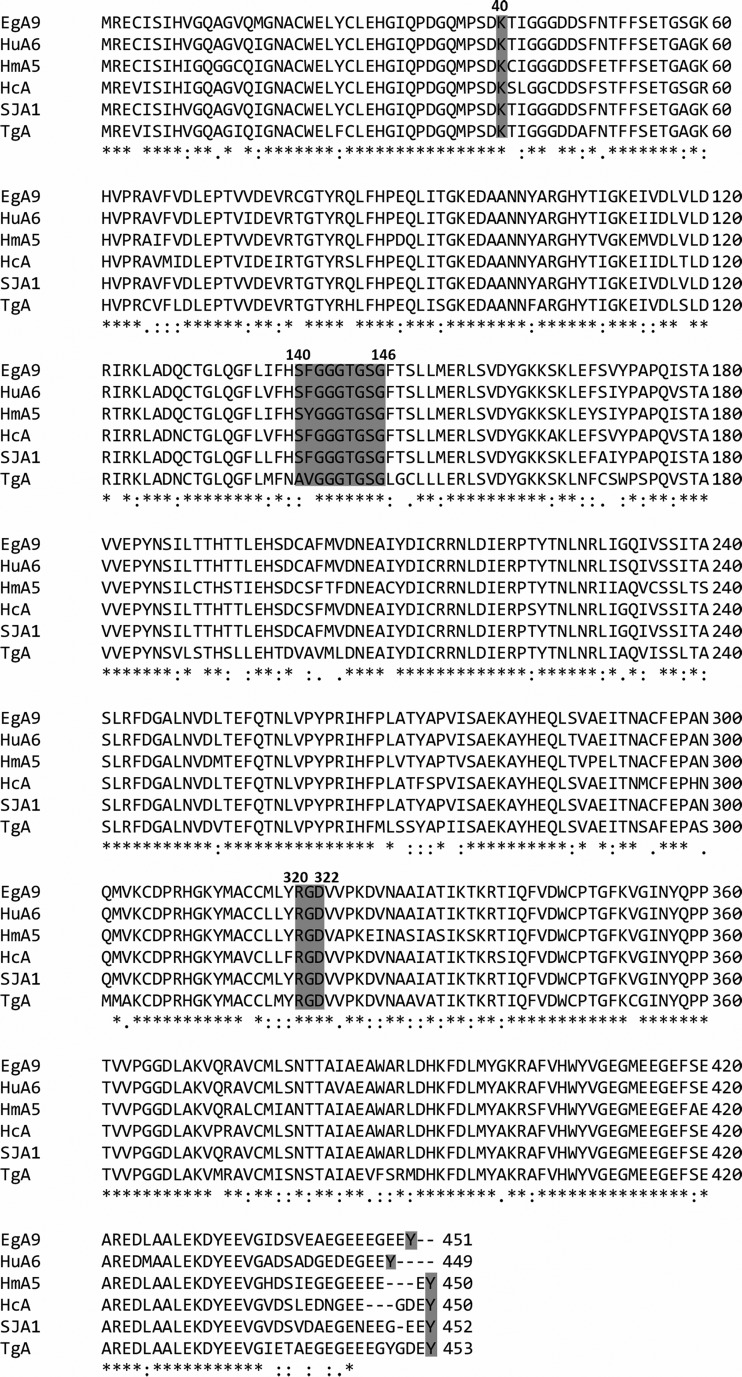
Sequence alignment of *Echinococcus granulosus* α_9_-tubulin and corresponding sequences from humans and parasites. The symbol “*” denotes the positions of amino acids that have a single, fully conserved amino acid residue; the symbol “:” denotes conservation between groups of amino acids with strongly similar properties; the symbol “.” denotes conservation between groups of amino acids with weakly similar properties; and the symbol “–” denotes gaps inserted to maximize sequence alignment. EgA9, *E. granulosus* α_9_; HuA6, human alpha 6 (119578461); HmA5, *H. microstoma* alpha 5 (674586714); HcA, *H. contortus* alpha tubulin (159155); SjA1, *S. japonicum* alpha 1–3 (226478902); and TgA, *T. gondii* alpha (161937).

**Figure 3.**
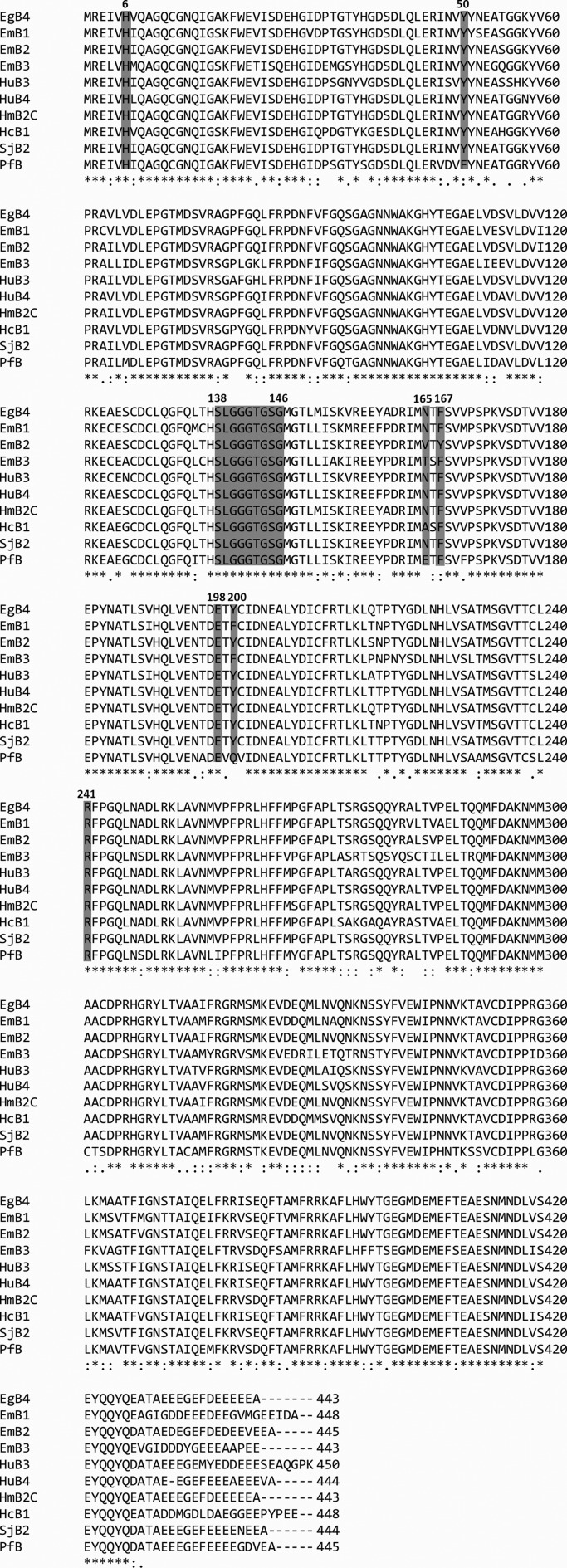
The sequence alignment of *Echinococcus granulosus* β_4_-tubulin and corresponding sequences from humans and parasites. Symbol meanings are as in Figure 2. EgB4, *E. granulosus* β_4_-tubulin; EmB1, *E. multilocularis* Tub-1 gene (7838198); EmB2, *E. multilocularis* Tub-2 gene (7838200); EmB3, *E. multilocularis* Tub-3 gene (7838202); HuB3, human B3 (50592996); HuB4, human B4a (574584803); HmB2C, *H. microstoma* beta 2C (674589300); HcB1, *H. contortus* beta tubulin isotype 1 (124244617); SjB2, *S. japonicum* beta 2 (226467271); PfB, *P. falciparum* beta (160732).

*Echinococcus granulosus* full-length α_9_- and β_4_-tubulin cDNAs were amplified; the amplicons contained 1356 bp and 1332 bp coding regions for α_9_- and β_4_-tubulin, respectively (Supplementary Table S1). The α_9_- and β_4_-tubulin genes were predicted to encode proteins with 451 and 443 amino acids, and the theoretical molecular masses were 50.17 kDa and 49.70 kDa (Figs. 1–3).

### Analyses of α_9_- and β_4_-tubulin sequences

**Figure 4.**
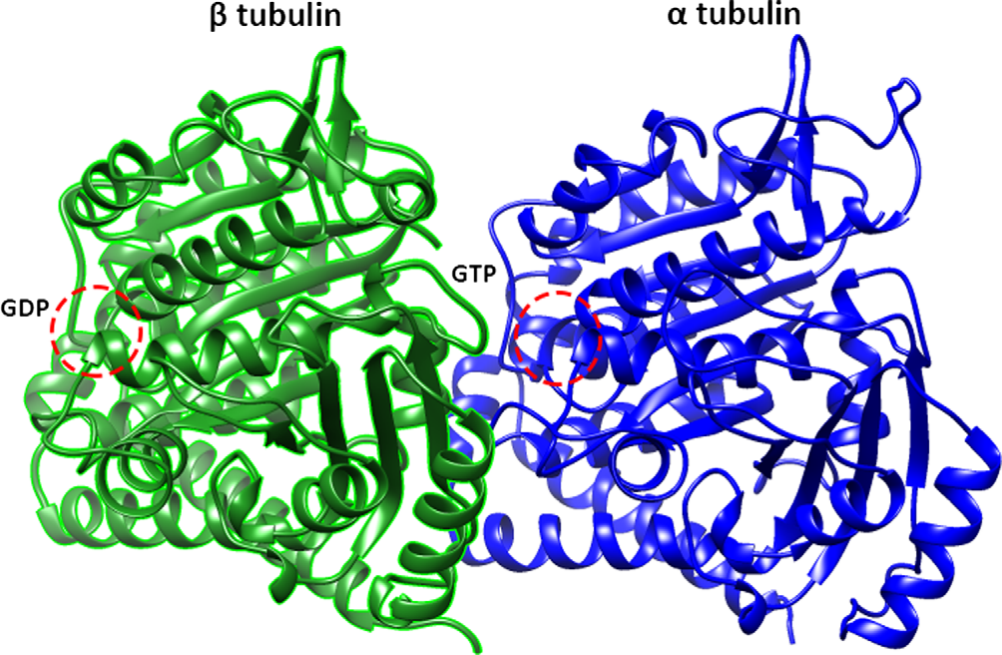
Modelled structure of the polymerized *Echinococcus granulosus* α_9_- and β_4_-tubulin dimer based on PDB ID 4f6r.

The sequences of *E. granulosus* α_9_- and β_4_-tubulin were compared with other α- and β-tubulins from different organisms, which showed high degrees of homology (Figs. 2 and 3), especially in some highly conserved domains. As shown in Figure 2, the conserved tubulin acetylation site K40 was also found in *E. granulosus* α_9_-tubulin and the α-tubulins of humans, *Hymenolepis microstoma*, *Haemonchus contortus*, *Schistosoma japonicum*, and *Toxoplasma gondii*. The potential GTP-binding site in *E. granulosus* α_9_-tubulin was present at residues 140–146 (Figs. 2 and 4). In addition, the RGD motif, serving as a cell attachment sequence, was located at residues 320–322. A tyrosine is conserved in highly divergent C-terminal sequences and is involved in the post-translation modifications (PTMs) of tyrosination/detyrosination.

Sequence alignment of *E. granulosus* β_4_-tubulin and β tubulins from other organisms indicated that *E. granulosus* β_4_-tubulin had conserved His6, Tyr50, Asn165, Ph167, Glu198, Tyr200, and Arg241 (Fig. 3). The potential GDP-binding site in *E. granulosus* β_4_-tubulin located at residues 138–146 was highly conserved in all groups (Figs. 3 and 4).

### Expression and purification of recombinant α_9_- and β_4_-tubulin

Recombinant α_9_/β_4_-tubulin was overexpressed mainly in inclusion bodies when *E. coli* BL21 (DE3) was induced with 1 mM IPTG. The purification yields of α_9_- and β_4_-tubulin were 2.0–3.0 mg/L and 3.5–5.0 mg/L of cell culture, respectively. Single protein bands with the expected molecular weight of α_9_-tubulin or β_4_-tubulin were found on SDS-PAGE gels (Fig. 1a). Furthermore, the recombinant protein was specifically recognized by commercial anti-His antibody, anti-α-tubulin antibody, and anti-β-tubulin antibody, which confirmed the successful expression of recombinant protein (Fig. 1b). The native α/β_-_tubulin of the *E. granulosus* protoscolex, which was in extremely low concentrations, was detected in Western blots by commercial anti-α- and anti-β-tubulin antibodies (Fig. 1b).

### Polymerization of recombinant α_9_- and β_4_-tubulin

**Figure 5.**
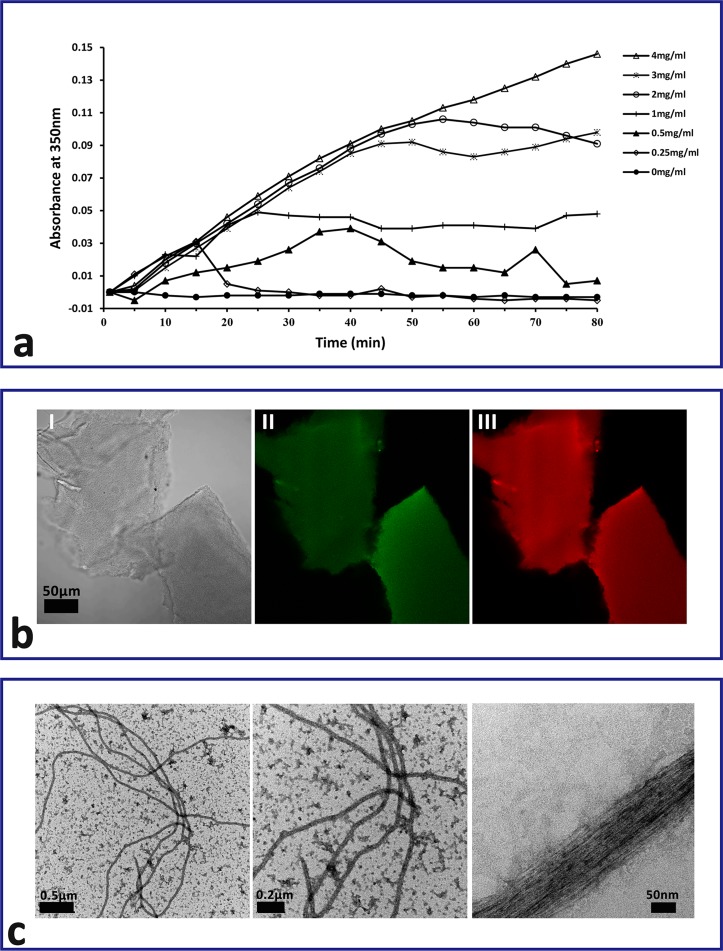
The polymerization of *Echinococcus granulosus* α_9_- and β_4_-tubulin. (a) Spectrophotometric analysis of polymerization of *E. granulosus* α_9_- and β_4_-tubulin at different concentrations. (b) Confocal scanning laser micrographs of the polymerization product of recombinant tubulin showing immune reactivity to anti-α-tubulin/Alexa-Fluor 488 antibody and anti-β-tubulin/Alexa-Fluor 647 antibody. (I): Phase contrast view, (II): α-tubulin immuno-reactivity, (III): β-tubulin immune-reactivity. (c) The microtubule-like structure resulting from polymerization of pure expressed α_9_- and β_4_-tubulin under appropriate conditions *in vitro*.

In this study, continuous A_350_ was recorded during polymerization of tubulin at different concentrations. An increase in absorbance was observed for the first 13–43 min, followed by a short initial lag period and a gradual levelling off (Fig. 5a). The optimum concentration of tubulin for polymerization was 2 mg/mL, and the curve is a typical polymerization curve that contains the nucleation, growth, and steady-state equilibrium phases of MT polymerization. By immunofluorescence, recombinant α_9_-tubulin and β_4_-tubulin were detected in polymerized tubulins (Fig. 5b), suggesting that these two tubulin isoforms could polymerize with each other under the proper conditions. In addition, the formation of a microtubule-like structure observed by electron microscopy again proved the polymerization of the tubulins (Fig. 5c).

## Discussion

MTs, which are built from α/β-tubulin heterodimers, play an important role in nearly all cellular and developmental processes of eukaryotic cells [23]. The *E. granulosus* α_9_- and β_4_-tubulin sequences reported in this study were retrieved from the publicly available *E. granulosus* genome data in the Sanger Database (https://www.sanger.ac.uk/resources/downloads/helminths/echinococcus-granulosus.html). There are 14 proteins denoted as α-tubulin and 10 proteins denoted as β-tubulin in the genome data. We denominated these as *E. granulosus* α_1_- to α_14_-tubulin and β_1_- to β_10_-tubulin and analyzed the transcript levels of these sequences by real-time PCR. In both the cyst and protoscolex, α_9_-tubulin and β_4_-tubulin were highly expressed (unpublished data). Hence, α_9_- and β_4_-tubulins were selected for subsequent studies.

Previous analyses of tubulin sequences indicated that tubulins are generally highly conserved among species, but the C-termini are highly divergent [6]. Unsurprisingly, some conserved sites and domains were also found in *E. granulosus* α_9_- and β_4_-tubulin, such as the acetylation sites [16], GTP-binding sites and the RGD sequence [11]. As the best-characterized acetylation site on tubulin, K40 was also conserved in α_9_-tubulin. Many studies have shown that MT acetylation is not necessary for cell survival [9] and is considered to be a marker of MT stability [31]. At present, the acetylation of MTs has mainly been studied in protozoans, and it was concluded that K40 acetylation stabilizes MTs and is required for parasite replication [36], but no data are available for *E. granulosus* or *E. multilocularis*. Moreover, the highly divergent C-terminal domain of tubulin is related to tubulin polymerization and interactions with other factors and proteins. The C-terminal sequence of *E. granulosus* α_9_-tubulin was not fully conserved from other α-tubulins, but the last conserved tyrosine residue indicated that *E. granulosus* α_9_-tubulin can undergo enzymatic removal and re-addition as part of a detyrosination/tyrosination cycle [41], which affects microtubule-associated proteins (MAPs) that function in a wide range of biological processes [25]. In this study, *E. coli* was used to produce sufficient amounts of tubulins for MT polymerization experiments, but the shortcoming of this expression system is the lack of protein modification. Therefore, the subsequent study of tubulin modification will require the use of a eukaryotic expression system.

In addition to the modification site, the drug binding sites of β-tubulin are of interest. Mutations at positions 6, 50, 165, 167, 198, 200 and 241 are related to benzimidazole resistance in parasites, fungi, and plants [2, 15, 29]. The sequence alignment indicated that His6, Tyr50, and Glu198 are conserved in most tubulins, as shown in Figure 3. In *H. contortus*, the F200Y mutation is most often related to the resistance profile. Specifically, helminths susceptible to benzimidazole present Phe at position 200; thus, replacing Phe with Tyr may confer the resistant phenotype [14]. Until now, no BZ resistance in *Echinococcus* spp. has been reported, but the reported analyses of *E. multilocularis* tubulin sequences predicted sensitivity of EmTub-1 and Em Tub-3 and low binding affinity of *Em* Tub-2 (Tyr200) for BZs. In our study, F200 of *E. granulosus* β_4_-tubulin was identical to that found in humans, *H. microstoma*, *H. contortus*, and *S. japonicum*, while this residue was Gln in *Plasmodium falciparum*. The substitution at position 200 by other amino acids indicated less drug toxicity for humans [18] and the weak effects of BZs on some protozoans [12, 34] and *Schistosoma* spp. [32]. Furthermore, the multiple isoforms of *E. granulosus* make the understanding of these key amino acids and domains more complex.

At a threshold concentration, tubulins can assemble into MTs *in vivo* and *in vitro* under certain conditions, including warm temperatures, a pH of 6.4–6.7, GTP, EGTA, Mg^2+^ and glycerol [27]. In recent decades, tubulin has been identified as a key target for antitumour drugs, anthelminthics, and fungicides [19, 42]. The tubulin stabilizers and destabilizers can be distinguished by observing the tubulin polymerization profile [10]. Until now, *in vitro* functional analyses have been performed using native tubulin purified from the mammalian brain, which is rich in tubulin, metazoan sources, *Tetrahymena thermophila*, kinetoplastid parasites (*Leishmania, Trypanosoma*) and *S. cerevisiae* [30]. However, there are still some drawbacks to the use of native tubulin. For example, the purified native tubulins were contaminated by their counterparts, such as MAPs and MT motor proteins [27]; furthermore, it is difficult to separate different tubulin isoforms, which may be non-uniformly distributed [21, 24]. In addition, the large-scale growth of kinetoplastid parasites makes it possible to harvest samples for purification of assembly competent tubulins, while the large-scale culture of *E. granulosus* or *E. multilocularis* free from host cells is difficult. Hence, the expression and purification of recombinant *E. granulosus* α_9_- and β_4_-tubulin was carried out in our study.

Western blotting analysis of total protein extracted from protoscoleces (Fig. 1b) showed that α- and β-tubulin concentrations were very low in parasites, which makes it technically challenging to isolate enough assembly-competent tubulin for *in vitro* studies. Early studies reported that recombinant tubulin was capable of polymerization into a microtubule-like structure [26, 37], which indicates that the high-yield recombinant tubulin can replace native tubulin for high-throughput experiments. Recombinant tubulins can be individually expressed in either a prokaryotic expression system or a eukaryotic expression system [26, 27, 37]. Recombinant neuronal human tubulin was expressed in SF9 cells, with the final yield of tubulin being 1 mg/L of culture [37], which is lower than that expressed in the prokaryotic expression system [26, 27]. In our study, two tubulin isoforms from the parasitic tapeworm *E. granulosus* were expressed in a prokaryotic expression system. The average yields of α_9_- and β_4_-tubulin were 2.0–3.0 mg/L and 3.5–5.0 mg/L of culture, respectively. SDS-PAGE analyses of induced bacteria cells demonstrated that α_9_- and β_4_-tubulins were all overexpressed mainly as inclusion bodies, which was consistent with other reports of parasite tubulins [11, 20, 27]. We previously optimized the induction conditions to maximize the solubility of recombinant proteins (α_9_- and β_4_-tubulins) in *E. coli* [40]. The optimized approaches include a low inducer concentration and a low cell cultivation temperature [33]. However, all these strategies still resulted in poor protein yield. To obtain sufficient amounts of tubulins for *in vitro* studies, the inclusion bodies should be washed and refolded in an appropriate buffer to harvest the active proteins, which is referred to as the urea-alkaline method [20, 22] and the on-column refolding method [11, 13]. In the present study, the inclusion bodies were collected, purified and refolded using nickel affinity chromatography, which is a modified version of methods developed by Jang and Kalme [11] and Koo *et al.* [13]. Moreover, it is a much simpler and more efficient way to harvest active proteins than using phosphocellulose [26, 27]. The purified recombinant α_9_- and β_4_-tubulins were of high purity (Fig. 1a) and free of other proteins [13]. TEM analysis showed that the purified tubulins were capable of polymerizing into microtubule-like structures using a standard dimerization assay, as previously reported [11, 13, 27]. The TEM results confirmed that purified recombinant tubulins could polymerize with typical long microtubule-like structures, which further implied that the modified techniques developed in our laboratory for purification of recombinant tubulin are effective and efficient. However, although the polymerization profile of α_9_- and β_4_-tubulins was demonstrated in our study, MTs were predicted to consist of multiple isoforms that could have distinct MT polymerization dynamics, functions, and interactions with MAPs and compounds. Hence, more research is needed in the future.

## Conclusions

In the present study, two tubulin isoforms were successfully cloned and purified and were capable of polymerizing into microtubule-like structures that can be used to screen anti-mitotic drugs, as reported in *H. contortus* [27]. In the future, these tubulins from *E. granulosus* can be used as a tool for high-throughput screening of new drugs or lead compounds against *E. granulosus*. In addition, it will be possible to observe whether the activity of BZs inhibits the polymerization of purified tubulin from *E. granulosus* to better understand the modes of action of these therapeutic compounds.

## Availability of data and materials

Supplementary Table S1 All data generated or analysed during this study are included in this published article and in the Supplemental file 1. Supplementary material is available at https://www.parasite-journal.org/10.1051/parasite/2018063/olm
